# Libman-Sacks Endocarditis Presenting as Acute Coronary Syndrome, Acute Heart Failure and Multiple Embolic Strokes

**DOI:** 10.7759/cureus.38849

**Published:** 2023-05-10

**Authors:** Sanchari Banerjee, Maham Ahmed, James Osei-Sarpong, Mostafa Vasigh, Dana Aiello

**Affiliations:** 1 Internal Medicine, State University of New York Upstate University Hospital, Syracuse, USA; 2 Cardiology, State University of New York Upstate University Hospital, Syracuse, USA

**Keywords:** libman-sacks endocarditis, systemic lupus erythematosus, acute heart failure, thromboembolism, acute coronary syndrome

## Abstract

Libman-Sacks endocarditis is a rare cardiovascular manifestation of systemic lupus erythematosus. It is described as sterile vegetative lesions which can damage heart valves resulting in complications such as acute coronary syndrome and heart failure and can embolize to cause cerebral and renal infarcts. We present the case of a young African American female presenting with pleuritic chest pain. She was initially admitted for acute coronary syndrome. She was later found to have severe mitral regurgitation and eventually received a transesophageal echocardiogram which confirmed the diagnosis of Libman-Sacks endocarditis. Her course was complicated with acute diastolic heart failure and several embolic strokes in the watershed anterior cerebral artery/middle cerebral artery (ACA/MCA) territories. She was started on anticoagulation and antiplatelet agents. Her underlying lupus was treated with immunosuppressive agents. This case demonstrates that a high index of suspicion for Libman-Sacks is crucial in patients with lupus if presenting with cardiovascular symptoms. Early and prompt diagnosis can prevent and lessen the many side effects associated with thromboembolism.

## Introduction

Libman-Sacks endocarditis (LSE) is a rare disease that is mostly found postmortem with a prevalence of about 0.9% to 1.6% [1]. It is characterized by aseptic, verrucous double-sided valvular lesions that are the most common cardiac presentation of systemic lupus erythematosus (SLE) [1]. While the majority are detected incidentally via echocardiography and can be overlooked due to its asymptomatic nature, symptomatic presentation ranges from mild valvular regurgitation to bacterial endocarditis and thromboembolic cerebrovascular events [1]. Cardiovascular involvement continues to be the leading cause of death for patients with SLE, warranting a thorough understanding of its ailment course to enable clinicians to recognize LSE early and prevent associated morbidity and mortality.

## Case presentation

**Figure 1:**
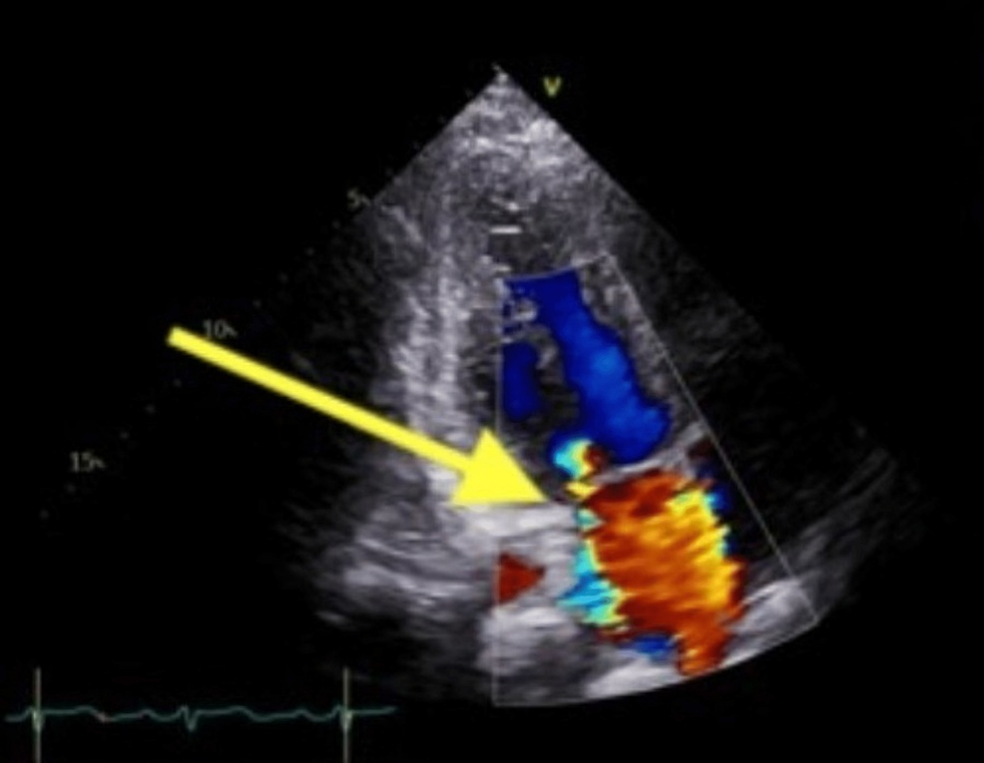
Transthoracic echocardiogram showing severe mitral regurgitation (yellow arrow) with a posteriorly directed jet of the posterior leaflet of the mitral valve

A 27-year-old African American female with a medical history significant for SLE presented with 24 hours of substernal pleuritic chest pain at rest radiating to her right arm. She also endorsed bilateral leg swelling and progressively worsening blurry vision for the past six months. Physical exam was significant for holosystolic murmur heard best at the mid-left to lower sternal border. Laboratory markers revealed elevated high sensitivity troponin from 1,719 to 4,629, acute kidney injury with a creatinine of 1.23 (baseline 0.7) and nephrotic range proteinuria and hematuria. EKG showed sinus rhythm with no acute ischemic changes. CT angiogram was negative for pulmonary embolism or dissection. Transthoracic echocardiogram showed an ejection fraction (EF) of 54% with hypokinesis of the basal mid and apical inferior wall, basal inferoseptum, mid-inferolateral wall and apical lateral wall. It also showed severe mitral regurgitation with a posteriorly directed jet of the posterior leaflet of the mitral valve (Figure 1). The patient was loaded with aspirin, clopidogrel, started on a heparin drip and admitted to the cardiac care unit for concern of non-ST elevation myocardial infarction (NSTEMI).

**Figure 2:**
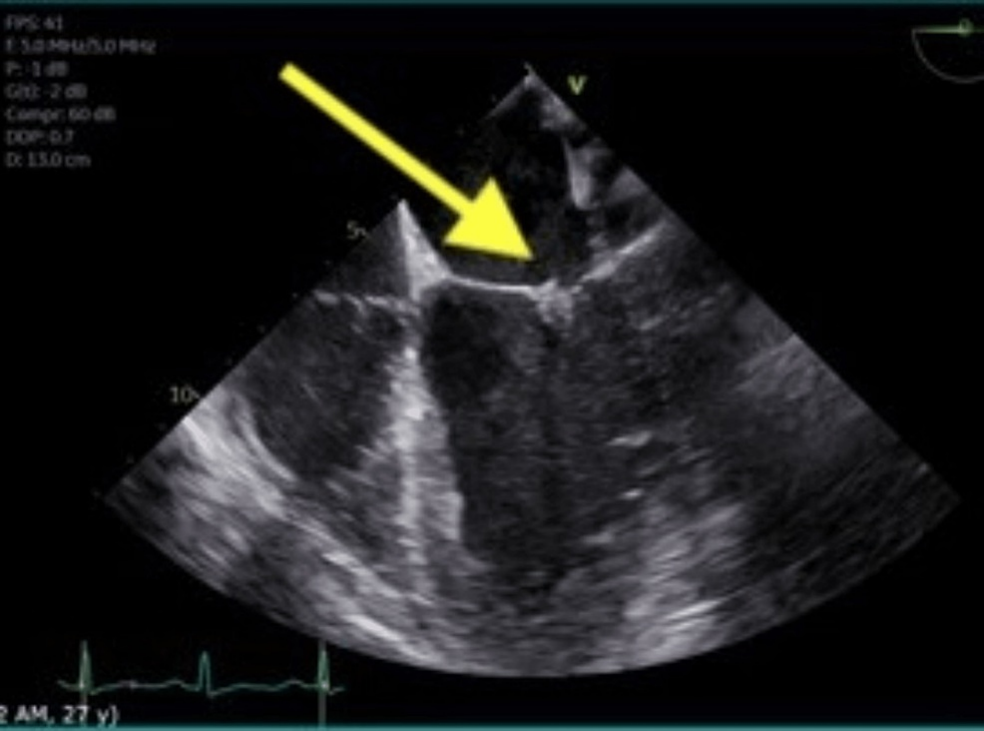
Transesophageal echocardiogram showing sessile densities (yellow arrow) of the mitral valve leaflet tips at the co-aptation points

**Figure 3:**
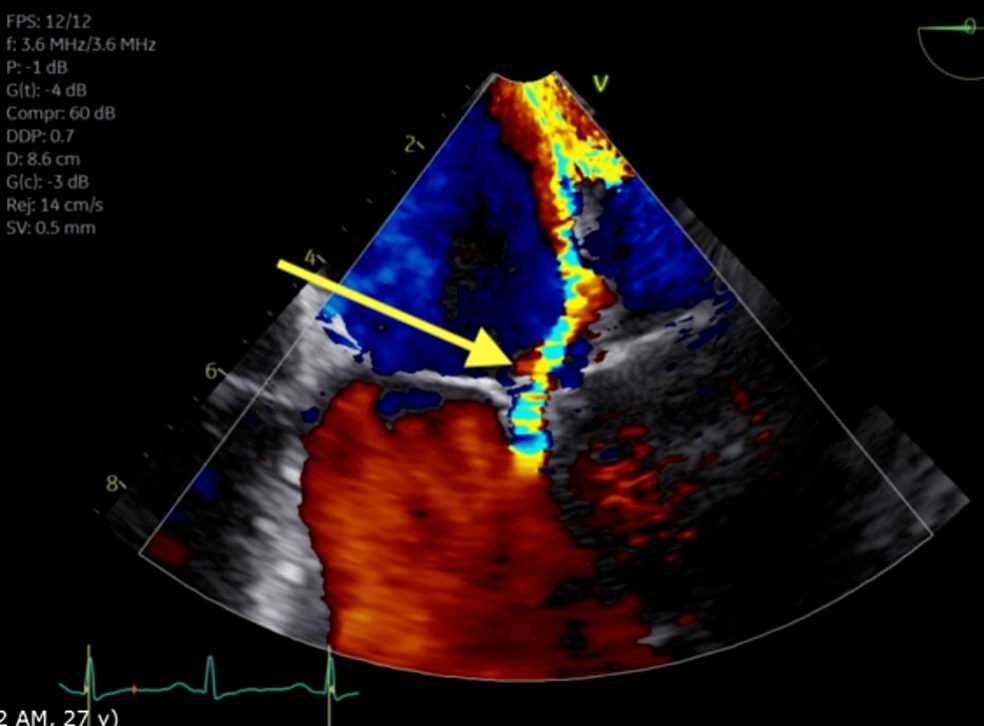
Transesophageal echocardiogram showing severe mitral regurgitation (yellow arrow)

She underwent left heart catheterization showing an occluded sub-branch of the posterolateral artery with left-to-left collaterals. There was a concern for possible spontaneous coronary artery dissection given the patient's age and coronary artery findings; hence, her occlusion was not stented. This was followed with a transesophageal echocardiogram (TEE) which showed severe mitral regurgitation and sessile densities of the mitral valve leaflet tips at the co-aptation points. There were no interatrial septum defects (Figures 2, 3). Blood cultures were negative. These findings were most consistent with LSE. She was started on anticoagulation. The surgical evaluation was obtained for mitral valve replacement, which was decided to be followed up outpatient.

**Figure 4:**
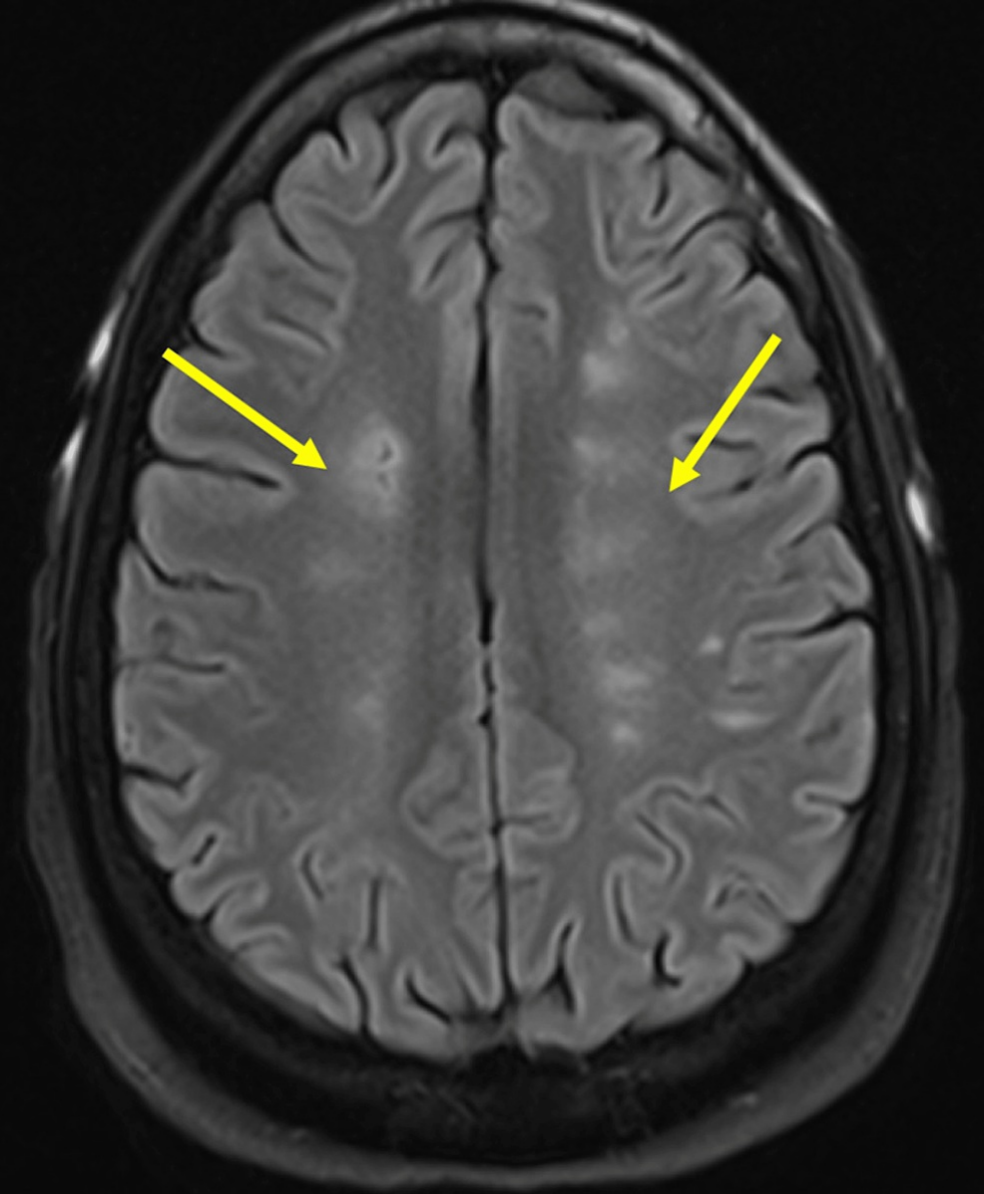
Axial flair sequences demonstrate areas of cystic encephalomalacia and surrounding gliosis (yellow arrows) along the watershed ACA/MCA territories distributed in a symmetric fashion in both cerebral hemispheres The findings are consistent with old ischemic changes.

**Figure 5:**
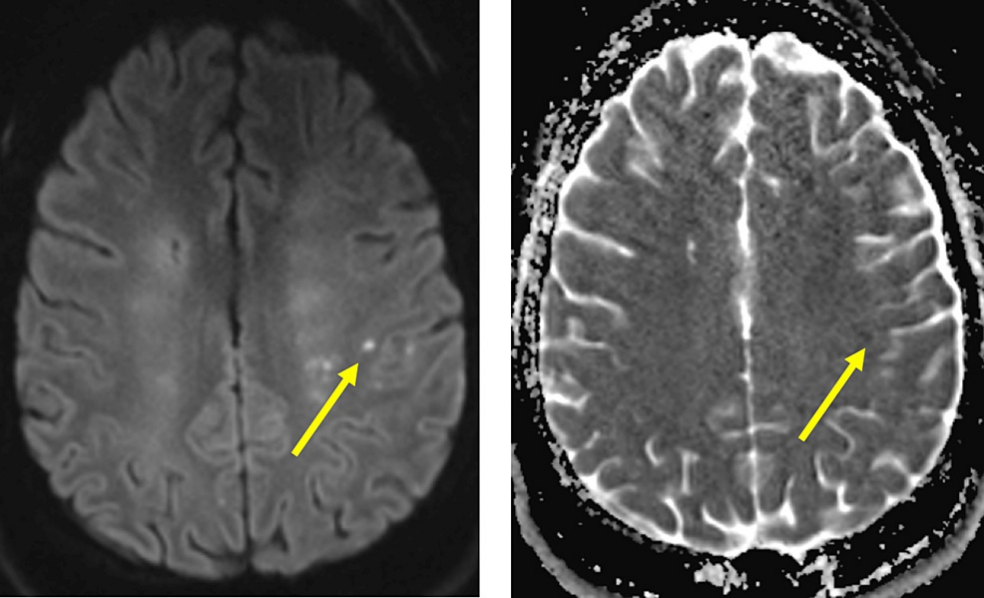
Small focus of T2 hyperintensity in the subcortical white matter in the left posterior frontal lobe (yellow arrows) which shows restricted diffusion on DWI/ADC mapping suggestive of an acute ischemic event, possibly embolic in etiology DWI/ADC: diffusion weight imaging/apparent diffusion coefficient.

Kidney biopsy revealed membranous lupus nephritis International Society of Nephrology (ISN)/Renal Pathology Society (RPS) Class V. Rheumatology recommended IV methylprednisolone 40 mg twice daily. Ophthalmology was consulted regarding her visual changes revealing optic disc edema, more prominent in the left eye. A follow-up magnetic resonance imaging (MRI) of the brain showed cystic encephalomalacia and surrounding gliosis along the watershed anterior cerebral artery/middle cerebral artery (ACA/MCA) territories distributed in a symmetric fashion in both cerebral hemispheres, concerning sequelae of remote ischemic changes (Figure 4)*. *There was also a small focus of T2 hyperintensity in the subcortical white matter in the left posterior frontal lobe suggestive of an acute ischemic event, possibly embolic in etiology (Figure 5)*. *Magnetic resonance venography did not show thrombosis. Anticardiolipin antibodies, both IgG and IgM, and anti-B2 glycoprotein antibodies as part of testing for antiphospholipid syndrome returned negative. Neurology deemed the patient to have had an acute ischemic stroke in the setting of cardioembolism from Libman-Sacks endocarditis. The patient refused lumbar puncture for evaluation of optic disc edema.

Throughout the course of her hospital stay, the patient developed acute decompensated heart failure and was diuresed with IV furosemide. She was stabilized and discharged on oral furosemide, low molecular weight heparin, clopidogrel, metoprolol, prednisone and atorvastatin with outpatient follow-up.

## Discussion

SLE is an inflammatory autoimmune disorder that affects mostly women between the ages of 15 and 44. Its incidence is highest in populations of African descent [2]. It can lead to the involvement of almost any organ system; however, cardiac manifestations involving the pericardium, myocardium, endocardium or coronary artery disease develop in more than 50% of lupus patients [3]. The pathogenesis of cardiac involvement is largely unknown with autoantibody development or autoimmune reactions being the most widely accepted hypotheses [1].

Libman-Sacks endocarditis is described to affect one in 10 SLE patients and is recognized as a non-infective endocarditis with verrucous vegetations on the valve leaflets or papillary muscles [1]. The most common valves affected are mitral followed by aortic, with regurgitation representing the predominant functional lesion. Heart failure, as seen in our patient, is often a sequela of severe valvular abnormalities [1]. Furthermore, the incidence of cardio-embolic cerebrovascular events in patients with LSE has been reported as 10%-20% [4].

Diagnosis is made primarily through transesophageal echocardiography which has greater sensitivity and specificity than transthoracic echocardiography [5,6]. Definitive diagnosis can be made pathologically by the demonstration of platelet thrombi on autopsy or surgical specimens [7]. The optimal treatment approach is poorly defined. Anticoagulation is recommended as secondary prevention for thromboembolic phenomena in patients who have had a thromboembolic event [3]. In cases of significant valvular dysfunction, surgical decisions should be according to established guidelines for valvular heart disease such as heart failure or acute valve rupture. Reports suggest that the prevention of recurrent embolization is the most common reason for surgery [8]. Patients with LSE should be closely followed up during treatment, as they can still develop thromboembolic phenomena while on anticoagulation.

## Conclusions

In conclusion, LSE is typically asymptomatic but should be strongly suspected when significant valve dysfunction develops during the course of SLE. If left untreated, it can lead to significant multi-organ complications including thromboembolic events. Greater awareness of these complications, close follow-up and careful cardiovascular examination are essential for earlier diagnosis and intervention.
